# Supplementary material for: An osteoinductive and biodegradable intramedullary implant accelerates bone healing and mitigates complications of bone transport in male rats
Source: Nat Commun. 2023 Jul 24;14:4455. doi: 10.1038/s41467-023-40149-5 (PMC10366099; doi:10.1038/s41467-023-40149-5)
Supplement: Supplementary file 1 — Supplementary Information [file 41467_2023_40149_MOESM1_ESM.pdf]

An osteoinductive and biodegradable intramedullary implant accelerates bone healing and mitigates complications of bone transport in male rats

Sien Lin[1†], Hirotsugu Maekawa[1†], Seyedsina Moeinzadeh[1], Elaine Lui[1,2], Hossein Vahid Alizadeh[1], Jiannan Li[1], Sungwoo Kim[1], Michael Poland[5], Benjamin C. Gadomski[5], Jeremiah T. Easley[6], Jeffrey Young[1], Michael Gardner[1], David Mohler[1], William Maloney[1], Yunzhi Peter Yang[1,3,4*]

[1]Department of Orthopaedic Surgery, School of Medicine, Stanford University; Stanford, CA 94305, USA

[2]Department of Mechanical Engineering, School of Engineering, Stanford University; Stanford, CA 94305, USA

[3]Department of Materials Science and Engineering, School of Engineering, Stanford University; Stanford, CA 94305, USA

[4]Department of Bioengineering, School of Medicine, Stanford University; Stanford, CA 94305, USA

[5]Orthopaedic Bioengineering Research Laboratory, Department of Mechanical Engineering, Colorado State University, Fort Collins, CO 80523, USA

[6]Preclinical Surgical Research Laboratory, Department of Clinical Sciences, Colorado State University, Fort Collins, CO 80523, USA

[†]These authors contributed equally to this work.

[*]Corresponding author. Email: ypyang@stanford.edu

Supplementary Information

Fig. S1. Microphotographs of PCL-TCP filament surface and the interface between hydrogel coating and PCL-TCP filament.
Fig. S2. Contact angle goniometry at the filament-coating interface.
Fig. S3. A customized 3D printed PCL-TCP device for evaluating the adhesion of the hydrogel-coated PCL-TCP scaffold made by HyTEC technique.
Fig. S4. Prolonged release of BSA or BMP-2 by applying additional PCL coatings onto the IM implant.
Fig. S5. The monolateral external fixation device designed for bone transport.
Fig. S6. The monolateral external fixation device in animal experiments.
Fig. S7. X ray results of dynamic bone healing in rat.
Fig. S8. 3D micro-CT images of femoral specimens on POD34 and POD55.
Fig. S9. 2D cross-sectional micro-CT images of femoral specimens on POD34 and POD55.

Fig. S10. Histology of the whole femoral specimens on POD34 or POD55.
Fig. S11. Histology of bone regeneration at implant area on POD34 or POD55.
Fig. S12. Bone remodeling at regenerate sites in the femoral specimens on POD34 or POD55.
Fig. S13. Expression of BMP-2 at docking sites or regenerate sites in the femoral specimens on POD34 or POD55.
Fig. S14. Expression of VEGF at docking sites or regenerate sites in the femoral specimens on POD34 or POD55.
Fig. S15. Expression of CD31 at docking sites or regenerate sites in the femoral specimens on POD34 or POD55.
Fig. S16. Typical appearances of pin-tract infection on POD34 or POD55 by visual inspection.
Fig. S17. Micro-CT quantification in peri-pin tract regions.
Fig. S18. Bacterial colony formation in agar gel pates.
Fig. S19. Bacterial species distribution identified by 16S next generation sequencing.
Fig. S20. Pathway affected by DO and IMI+B2 during the early stage of bone regeneration.
Fig. S21. Representative photos and radiographs of the sheep metatarsal bone transport model, distraction and device implantation.
Table S1. Animal groups, treatments and the assessments in the study using rat bone transport model.
Table S2. Pin tract infection details evaluated accordingly to a modified Checketts classification method by visual inspection.

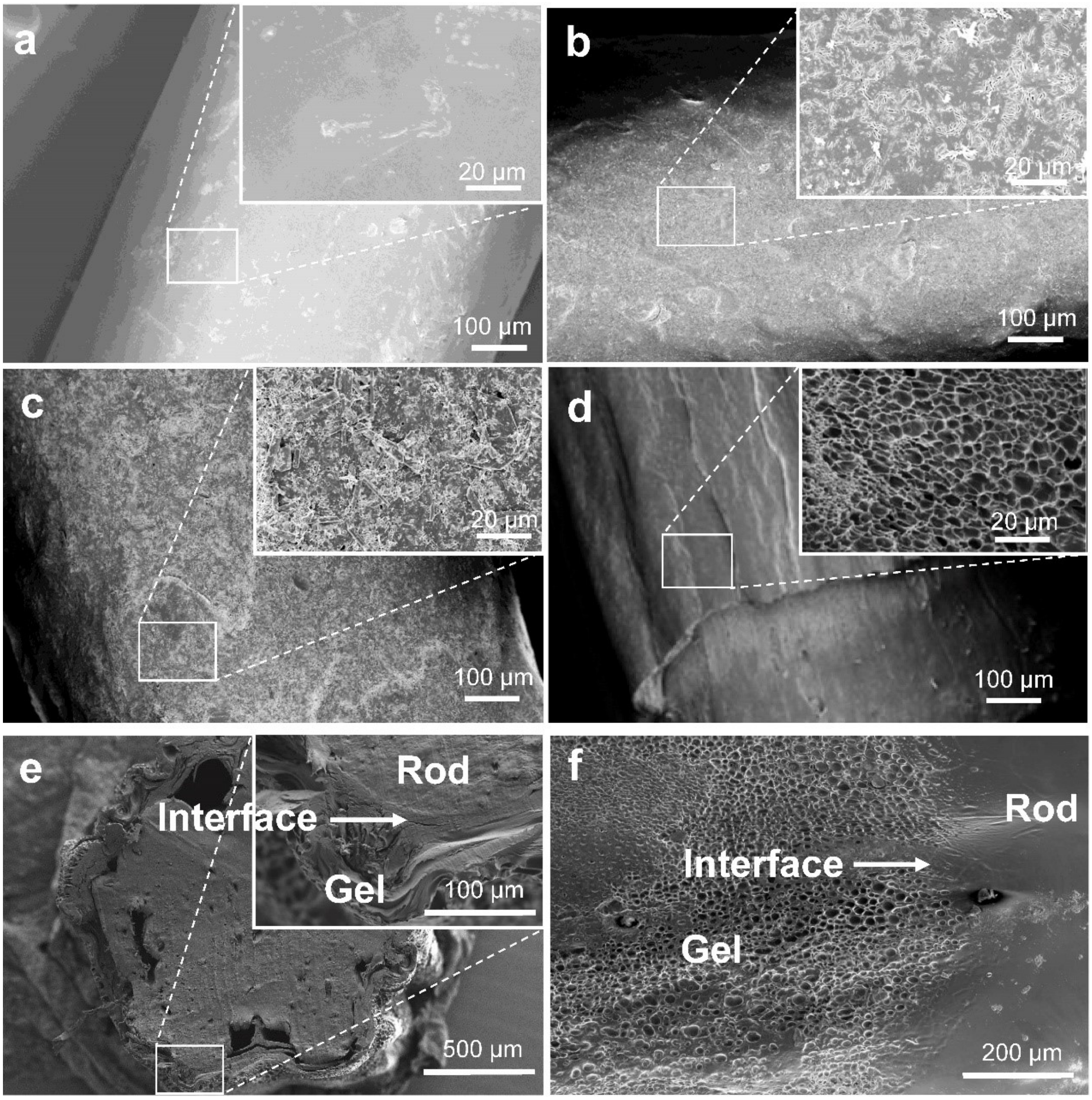

Fig. S1. Microphotographs of PCL-TCP filament surface and the interface between hydrogel coating and PCL-TCP filament. (a) Scanning electron microscopy (SEM) images of PCL-TCP filaments before any treatments; (b) after NaOH and freezing/thawing; (c) after $CaSO_4$ deposition onto the surface; (d) after coated with composite hydrogel and freeze-dried. (e) Cross-sectional or (f) longitudinal hydrogel-coated PCL-TCP filaments, showing a tight adhesion and smooth hydrogel-filament interface. Inserted images showing higher magnifications. This *ex vivo* characterization was repeated at least two times independently.

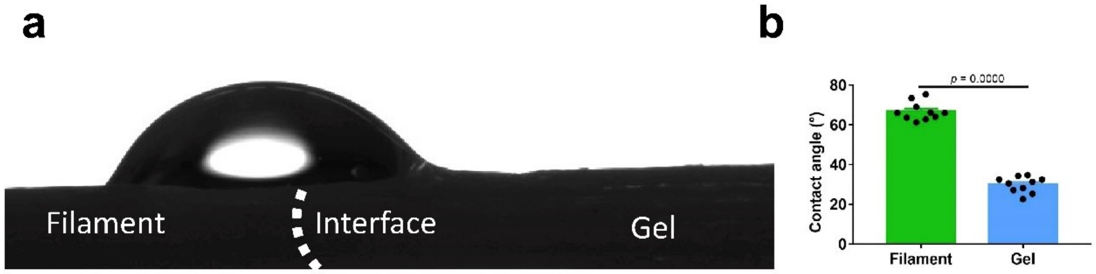

Fig. S2. Contact angle goniometry at the filament-coating interface. (a) Water droplet (2 μL) centered at the interface of the PCL-TCP filament and Hytec gel coating. (b) Contact angle measurements of the filament and gel. Data are represented as a bar graph with mean and s.e.m. values (n = 10 independent samples per group). Source data are provided as a Source data file.

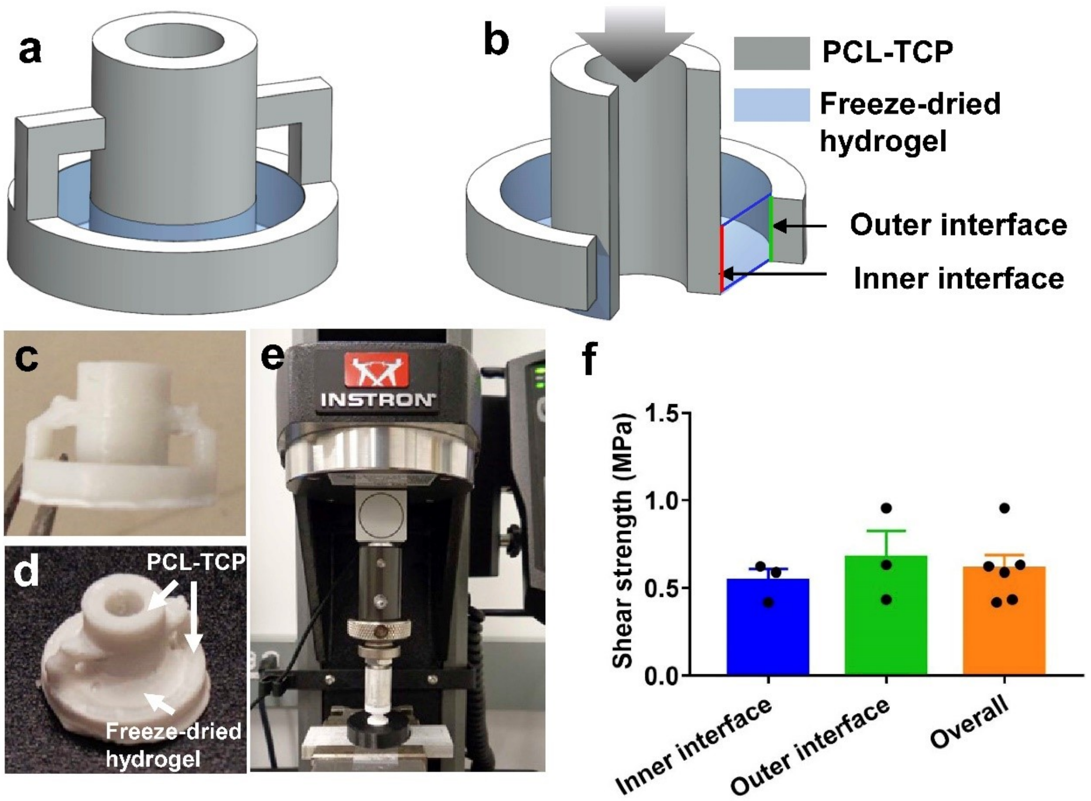

Fig. S3. A customized 3D printed PCL-TCP device for evaluating the adhesion of the hydrogel-coated PCL-TCP scaffold made by HyTEC technique. (a) A schematic of the 3D printed PCL-TCP device which was used to measure the adhesion of freeze-dried composite hydrogels to scaffolds. The device composed of two concentric cylinders connected through two bridges. (b) A schematic of the shear test experiment with cross sectional view of the device. Freeze-dried hydrogel was filled between the inner and outer ring. The top arrow represents the force applied to the device during the test. (c) The 3D printed device before loading hydrogel. (d) The device with freeze-dried hydrogel within the gap between two concentric cylinders. (e) Experimental set up to measure interfacial shear strength. (f) Six samples were tested, where three samples were observed to break at the inner ring interface, and three samples were observed to break at the outer ring interface. The overall interfacial shear strength of 0.609 ± 0.194 MPa was obtained by considering combined inner and outer interfacial strength measurements. Data are represented as a bar graph with mean and s.e.m. values (n = 3 independent samples per group). Source data are provided as a Source data file.

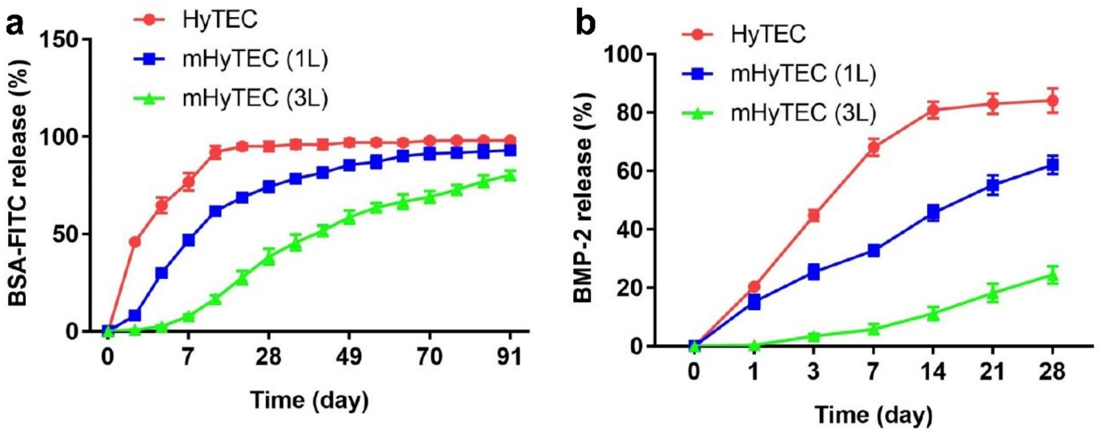

Fig. S4. Prolonged release of BSA or BMP-2 by applying additional PCL coatings onto the IM implant. A modified HyTEC (mHyTEC) technique using a single- (1L) or triple-layer (3L) of PCL coating approach has been applied. (a) Release kinetics of BSA from the HyTEC and mHyTEC grafts with one or three PCL layers for 91 days or 13 weeks. (b) Release kinetics of BMP2 from the HyTEC and mHyTEC grafts with one or three PCL layers for 28 days. Data are presented as curves with mean and s.e.m. values (n = 3 independent samples per group). Source data are provided as a Source data file.

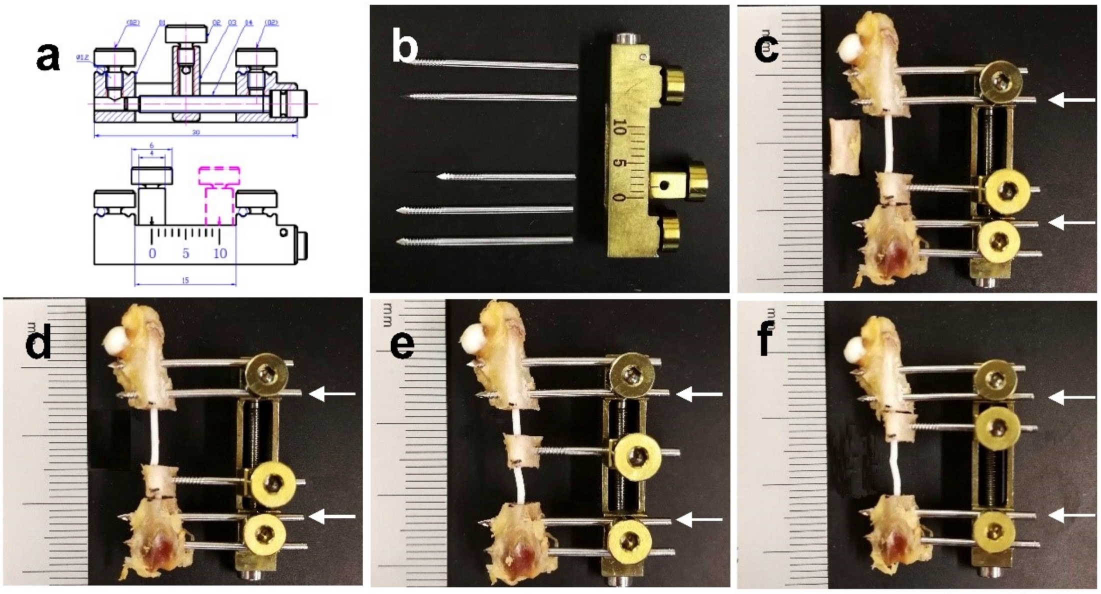

Fig. S5. The monolateral external fixation device designed for bone transport. (a) Schematic design of the fixator frame. (b) The customized external fixation device consists of two parts: one frame and five fixative pins. (c to f) Ex vivo demonstration of the device in bone transport over an IM implant using an adult rat femoral specimen after osteotomies. The implant was fixed by the two fixative pins in a press-fit (arrows).

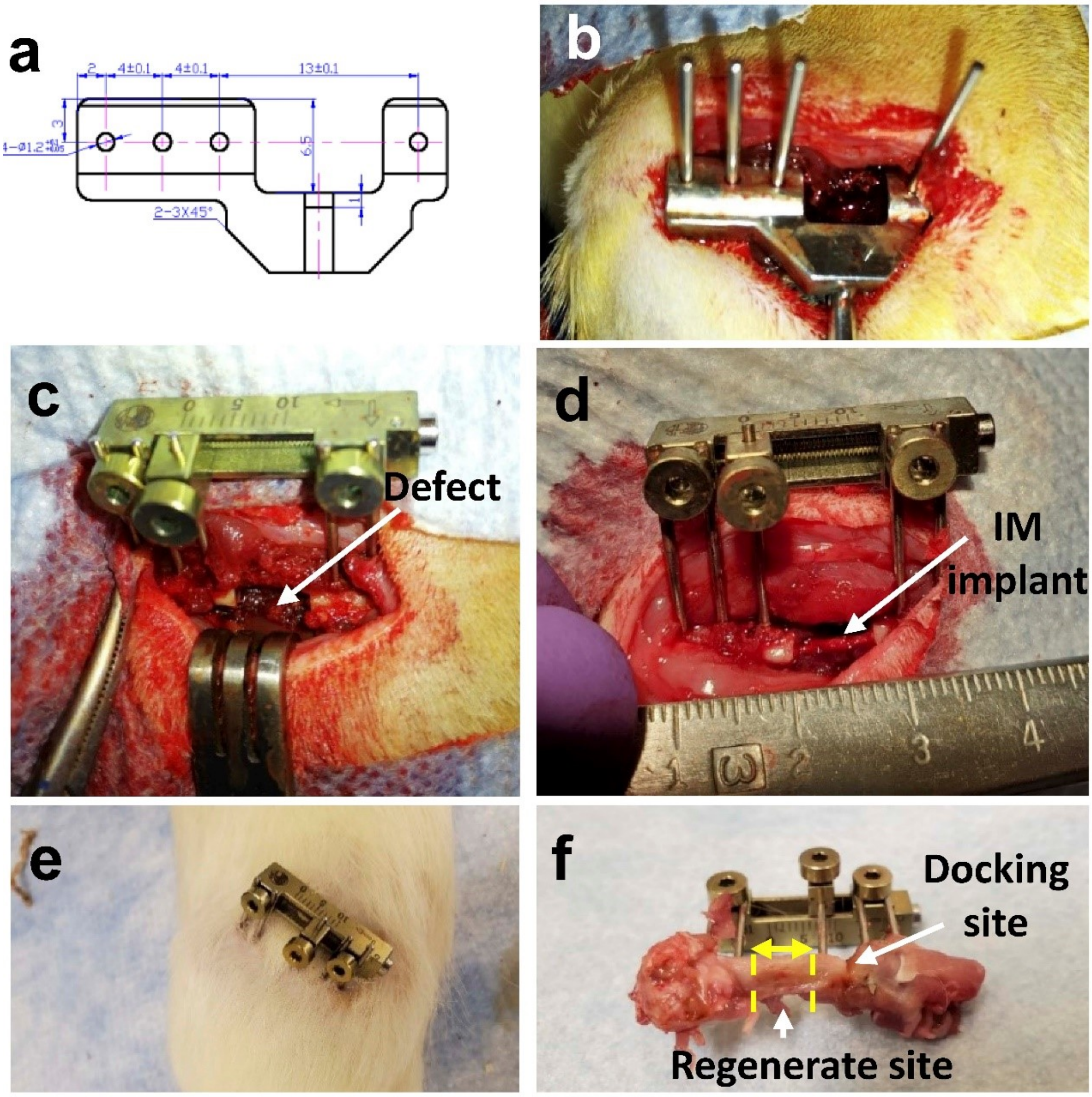

Fig. S6. The monolateral external fixation device in animal experiments. (**a**) Schematic design of the drill guide for pin fixation. (**b**) The drill guide used during operation. (**c**) Bone defect created after osteotomy by wire saw. (**d**) IM implant fixed intramedullary in the defect site. (**e**) Normal appearance of fixation device on a rat. (**f**) One representative femoral sample with fixation device after harvested.

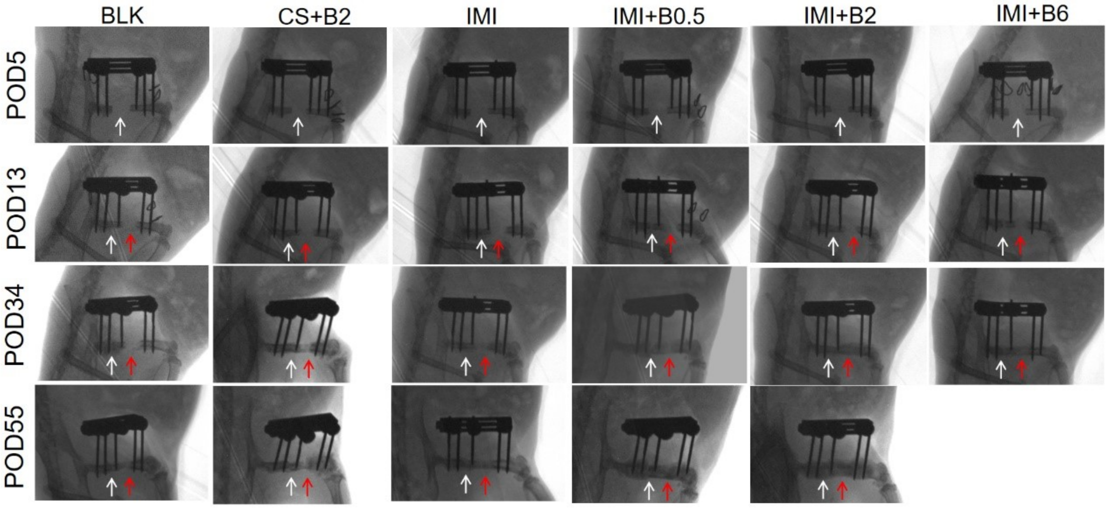

Fig. S7. X ray results of dynamic bone healing in rat. The X-ray imaging was conducted on POD5 (before distraction), POD13 (after distraction), POD34 (3-week consolidation), and POD55 (6-week consolidation). White and red arrows indicate the docking site and regenerate site, respectively.

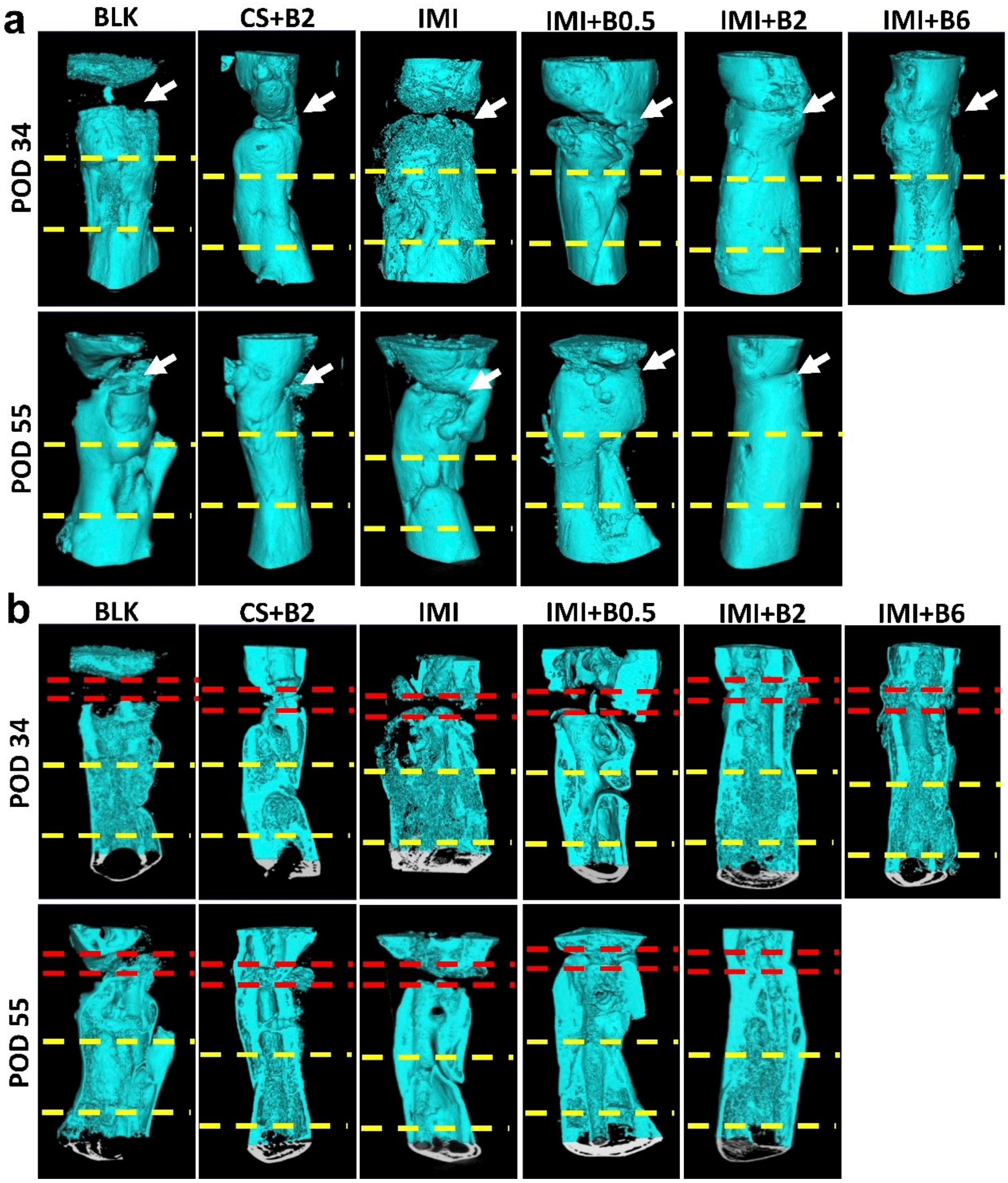

Fig. S8. 3D longitudinal micro-CT images of femoral specimens on POD34 and POD55. (a & b) Total or center longitudinal view of bone regeneration at regenerate sites (between yellow dashed lines) and docking sites (arrows or between red dashed lines).

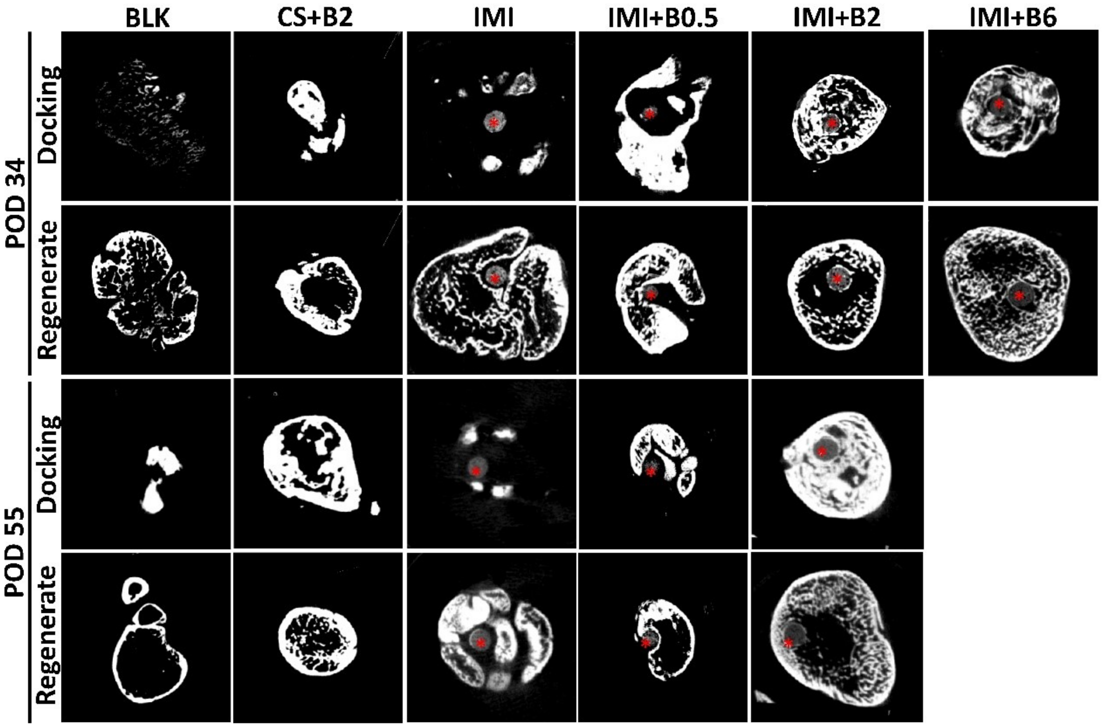

Fig. S9. 2D cross-sectional micro-CT images of femoral specimens on POD34 and POD55. Cross-sectional images showing bone regeneration showing bone regeneration at regenerate sites and docking sites with or without implants (asterisk) on POD34 and POD55.

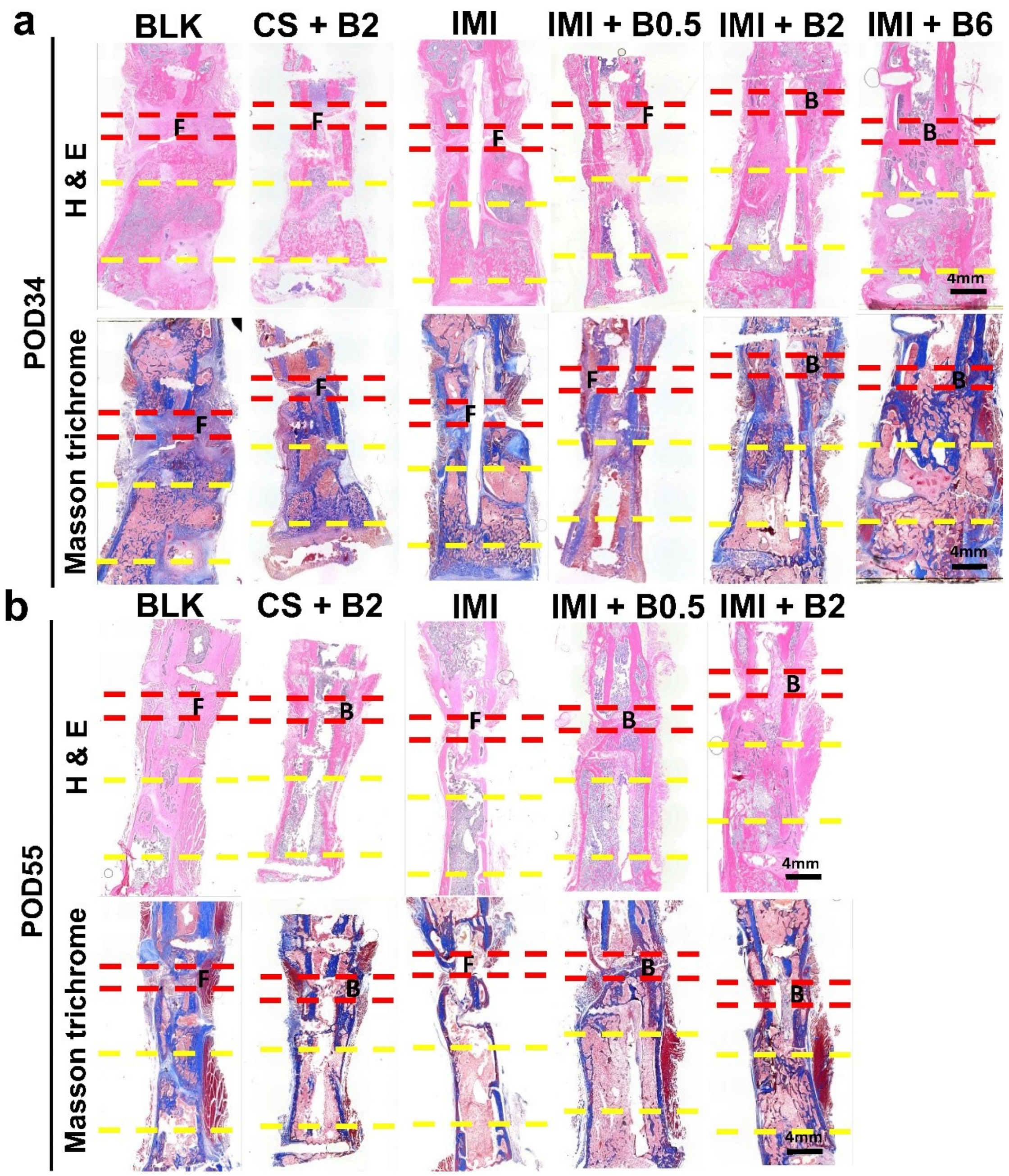

Fig. S10. Histology of the whole femoral specimens on POD34 or POD55. (a) Representative microphotographs on POD 34. (b) Representative microphotographs on POD 55. Longitudinal sections were stained by hematoxylin and eosin (H&E) or Masson trichrome. F, fibrous tissue; B: regenerated bone. Histological staining was conducted three times independently.

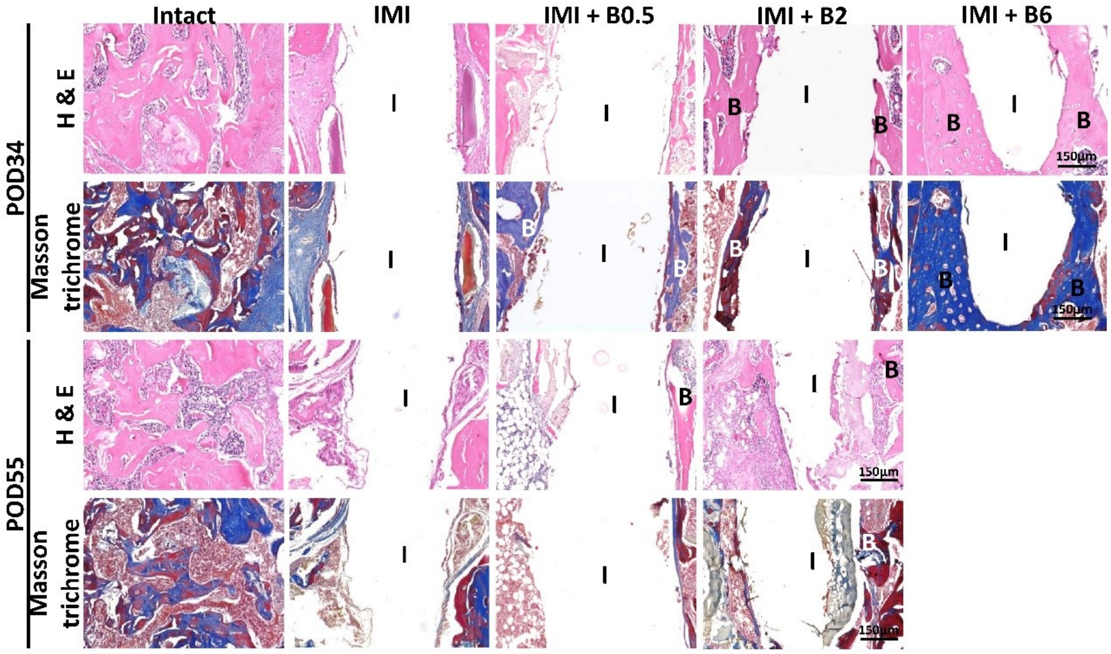

Fig. S11. Histology of bone regeneration at implant area on POD34 or POD55. Longitudinal sections were stained by hematoxylin and eosin (H&E) or Masson trichrome. I, implant; B: regenerated bone. Histological staining was conducted three times independently.

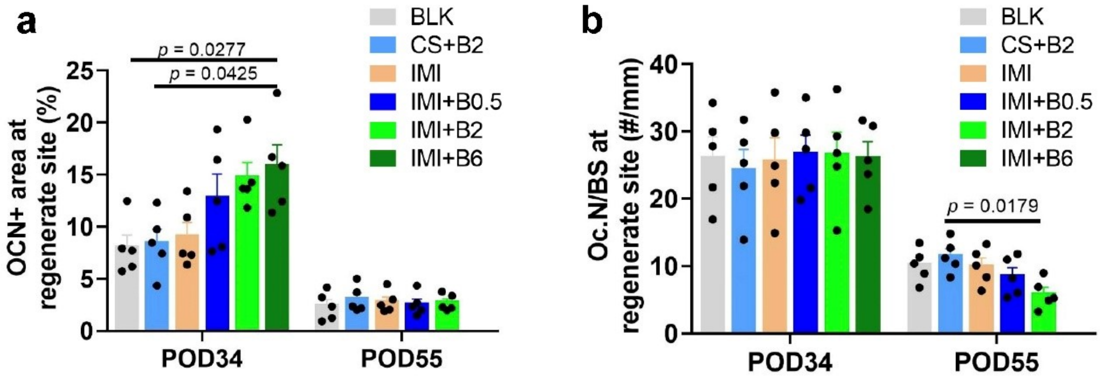

Fig. S12. Bone remodeling at regenerate sites in the femoral specimens on POD34 or POD55. (a) Semi-quantitative results of OCN positive expression area at regenerate sites. (b) Semi-quantitative results of TRAP-positive osteoclasts numbers per bone surface (Oc.N/BS) at the regenerate sites. Data are presented as scatter dot plot with mean and s.e.m. values (n = 5 independent rats per group). $^{*}P < 0.05$. One-way ANOVA and Tukey's multiple comparisons test. Source data are provided as a Source data file.

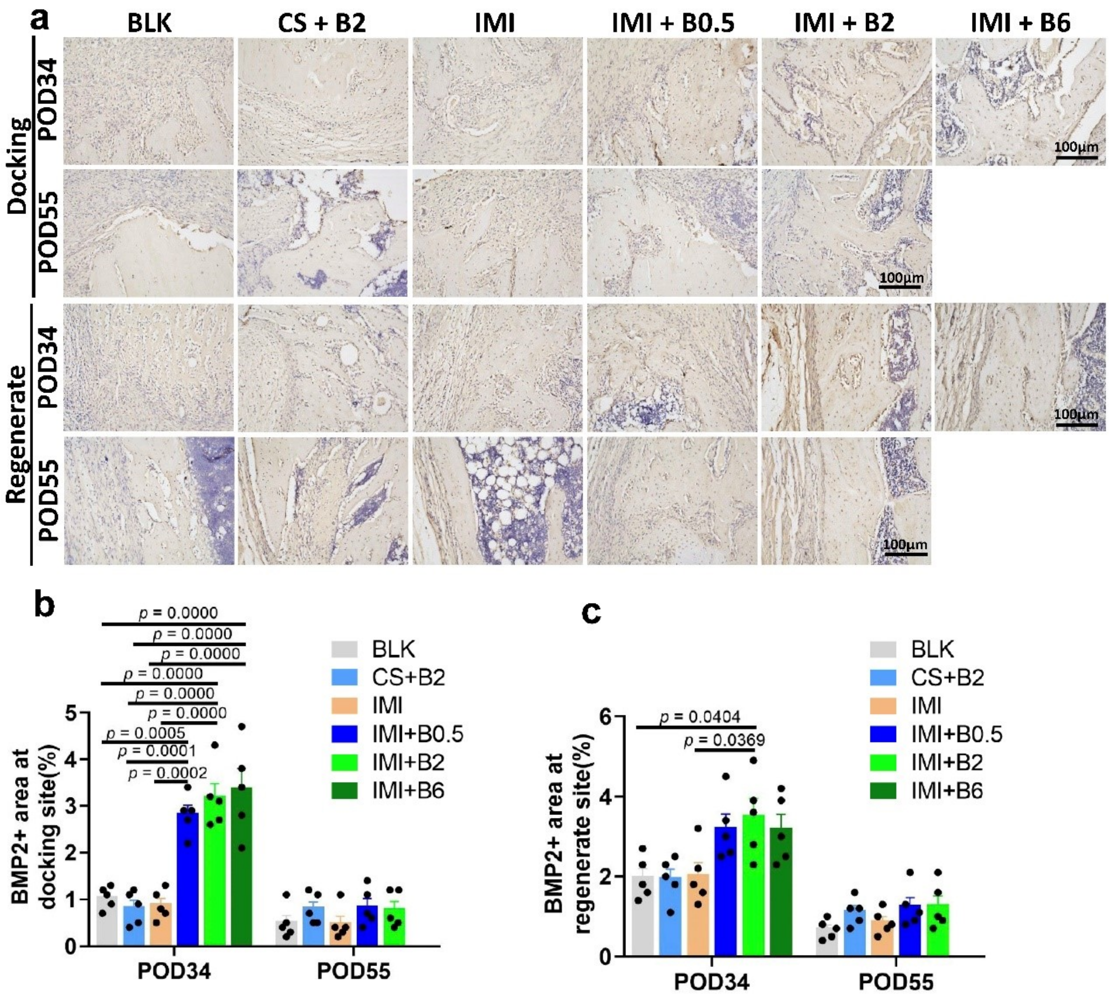

Fig. S13. Expression of BMP-2 at docking sites or regenerate sites in the femoral specimens on POD34 or POD55. (a) Osteogenic marker (BMP-2) was stained by immunohistochemistry. (b) Semi-quantitative results of BMP-2 at docking site. (c) Semi-quantitative results of BMP-2 at regenerate site. Data are presented as scatter dot plot with mean and s.e.m. values (n = 5 independent samples per group). $^{*}P < 0.05$. One-way ANOVA and Tukey's multiple comparisons test. Source data are provided as a Source data file.

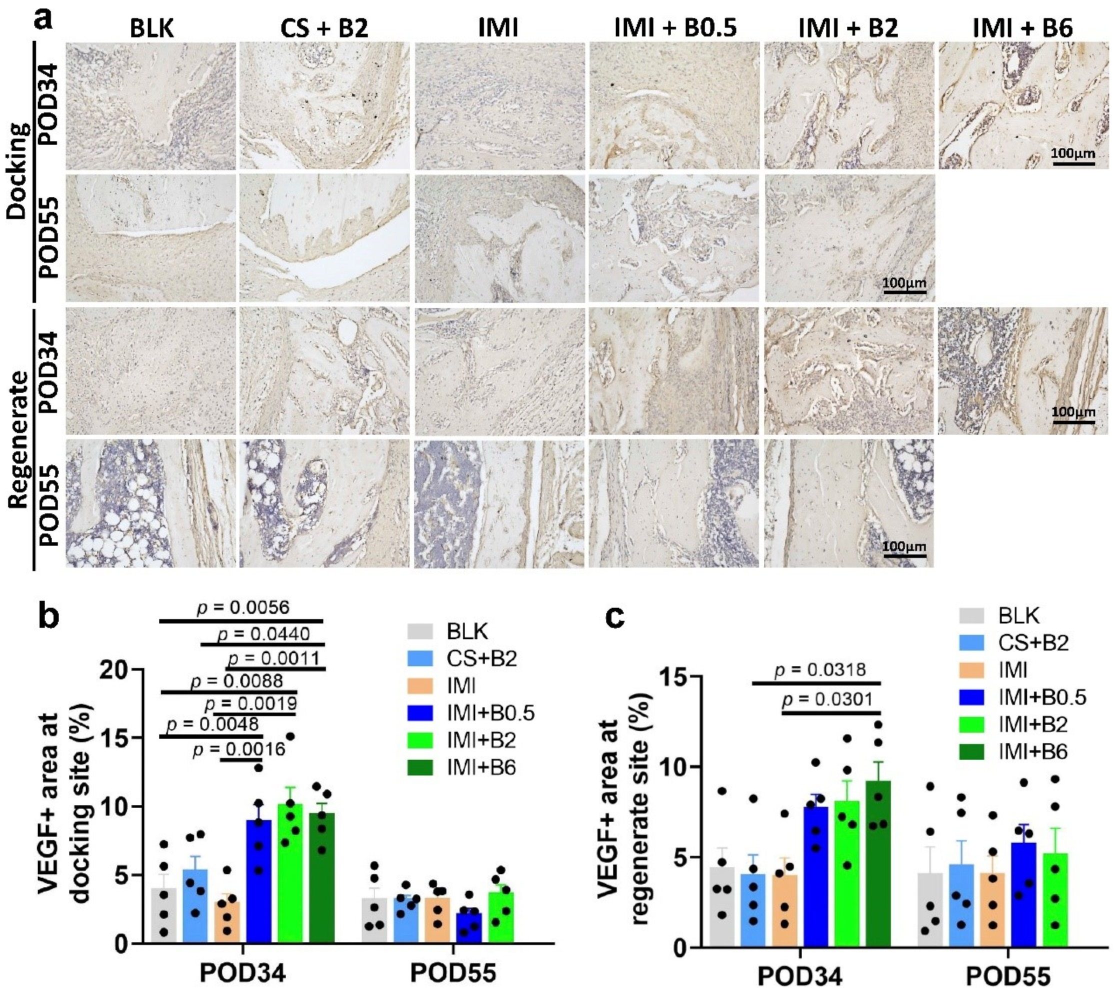

Fig. S14. Expression of VEGF at docking sites or regenerate sites in the femoral specimens on POD34 or POD55. (a) Angiogenic marker (VEGF) was stained by immunohistochemistry. (b) Semi-quantitative results of VEGF at docking site. (c) Semi-quantitative results of VEGF at regenerate site. Data are presented as scatter dot plot with mean and s.e.m. values (n = 5 independent samples per group). $^{*}P < 0.05$, $^{**}P < 0.01$, $^{***}P < 0.001$. One-way ANOVA and Tukey's multiple comparisons test. Source data are provided as a Source data file.

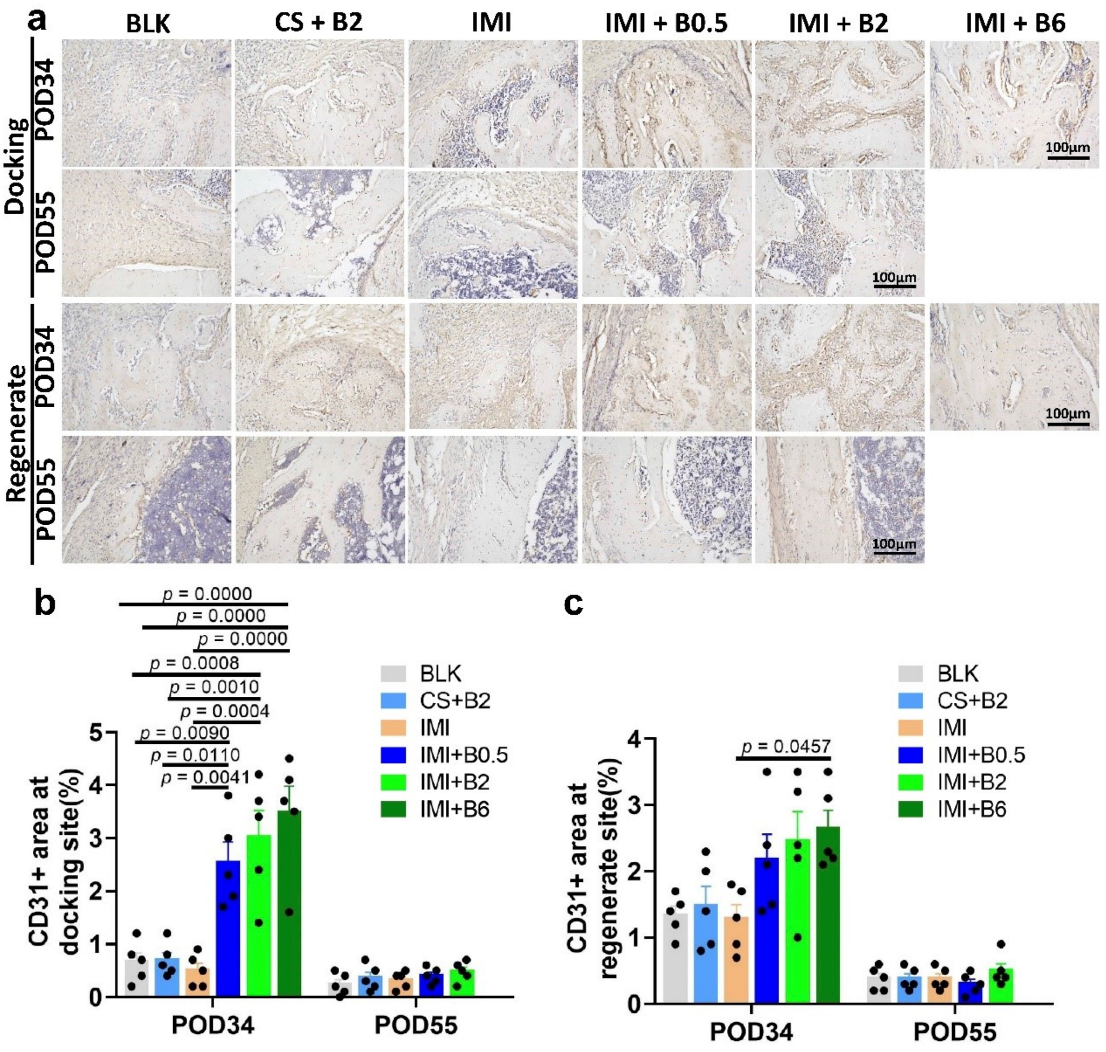

Fig. S15. Expression of CD31 at docking sites or regenerate sites in the femoral specimens on POD34 or POD55. (a) Vascularization marker (CD31) was stained by immunohistochemistry. (b) Semi-quantitative results of CD31 at docking site. (c) Semi-quantitative results of CD31 at regenerate site. Data are presented as scatter dot plot with mean and s.e.m. values (n = 5 independent samples per group). $^{*}P < 0.05$, $^{**}P < 0.01$, $^{***}P < 0.001$. One-way ANOVA and Tukey's multiple comparisons test. Source data are provided as a Source data file.

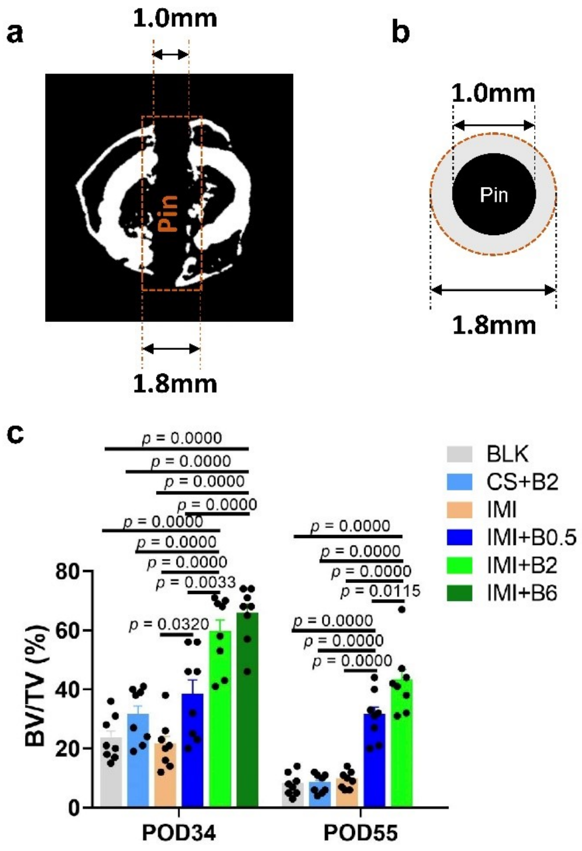

Fig. S16. Micro-CT quantification in peri-pin tract regions. (a) Region of interest (ROI) perpendicular to the long axis of the pin tract for quantification of peri-pin tract by Micro-CT analysis. (b) Schematic cross-sectional area of the pin tracts illustrating the ROI. (c) Bone volume/ tissue volume (BV/TV) of peri-pin tract region on POD34 and POD55. One-way ANOVA and Tukey's multiple comparisons test were applied in the analysis of micro-CT data. Data are presented as scatter dot plot with mean and s.e.m. values (n = 8 independent samples per group). $^{*}P < 0.05$, $^{**}P < 0.01$, $^{***}P < 0.001$. One-way ANOVA and Tukey's multiple comparisons test. Source data are provided as a Source data file.

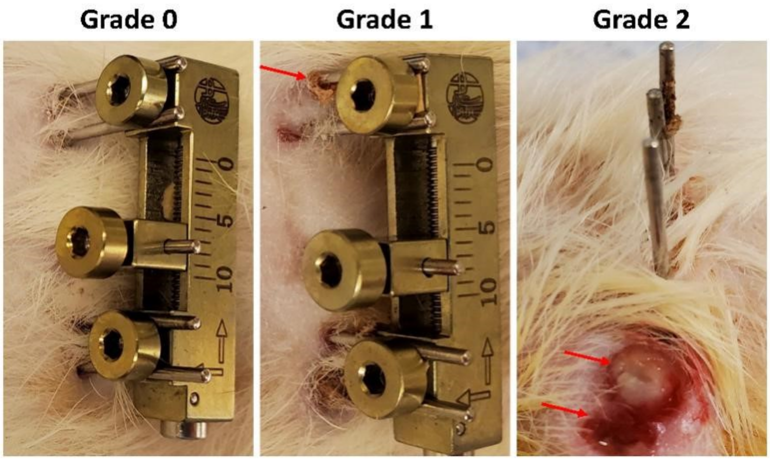

Fig. S17. Typical appearances of pin-tract infection on POD34 or POD55 by visual inspection. Grade 0: No redness, discharge, or pin loosening ; Grade 1: Redness and discharge around the pin but no pin loosening ; Grade 2: Redness and discharge around the pin, with pin loosening due to osteomyelitis. Red arrows point to the infection sites.

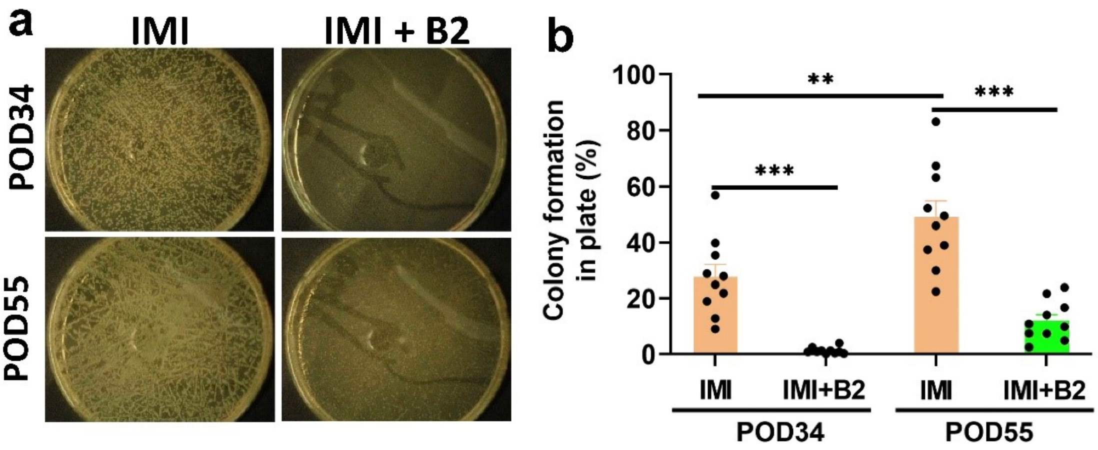

Fig. S18. Bacterial colony formation in agar gel pates. Bacteria cultured in Terrific Broth (TB) medium for 16 hours and later in agar gel plates for another 16 hours were isolated from pins after removal in IMI and (IMI + B2) groups on POD34 or POD55 (2 rats with 10 pins per time point per group), respectively. (**a**) The colony formation was examined after the TB medium (30 µl) added onto the agar gel plates and incubated for 16 hours in 37°C incubator. (**b**) Colony formation positive area was determined by the colony area/ total area in the plate. Data are presented as scatter dot plot with mean and s.e.m. values (n = 10 independent samples per group). One-way ANOVA and Tukey's multiple comparisons test. Source data are provided as a Source data file.

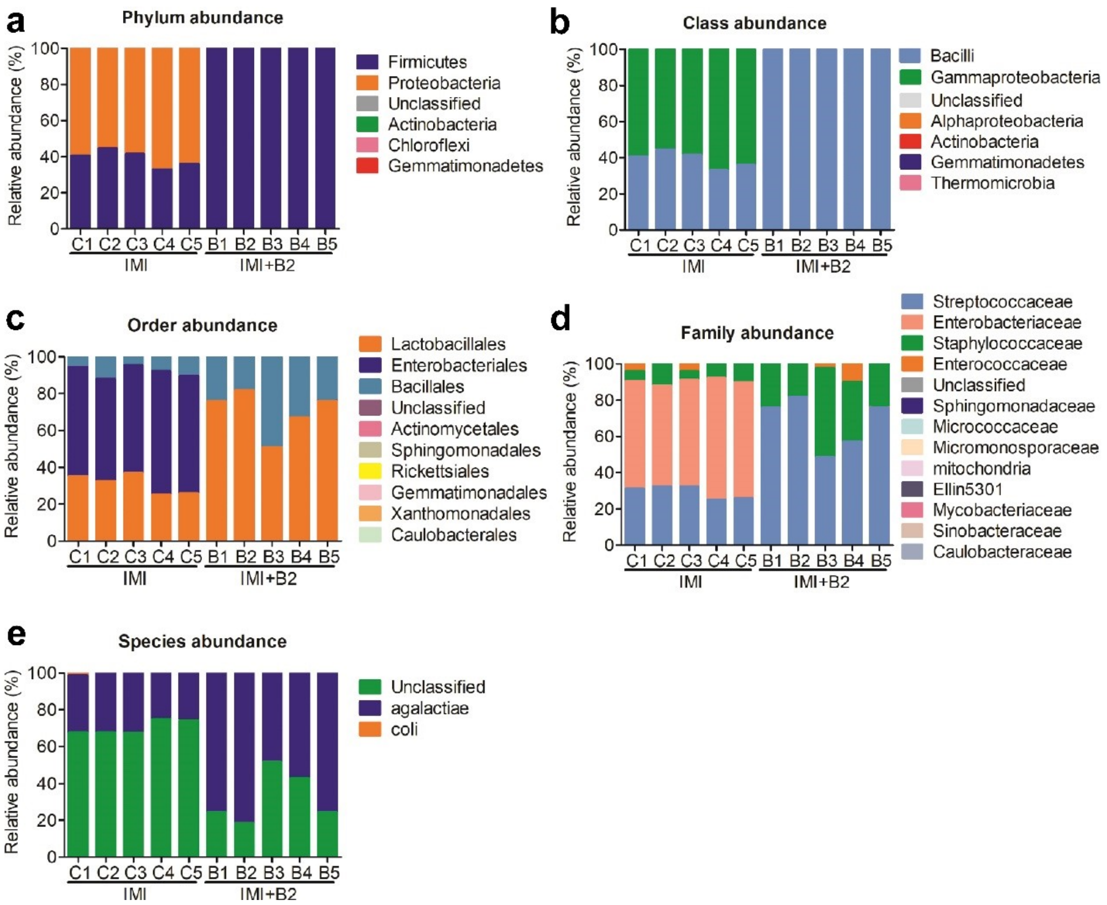

Fig. S19. Bacterial species distribution identified by 16S next generation sequencing. The distribution of the top 30 most abundant classifications in each sample or group at different taxonomic levels including (a) Phylum abundance, (b) Class abundance, (c) Order abundance, (d) Family abundance, and (e) Species. Source data are provided as a Source data file.

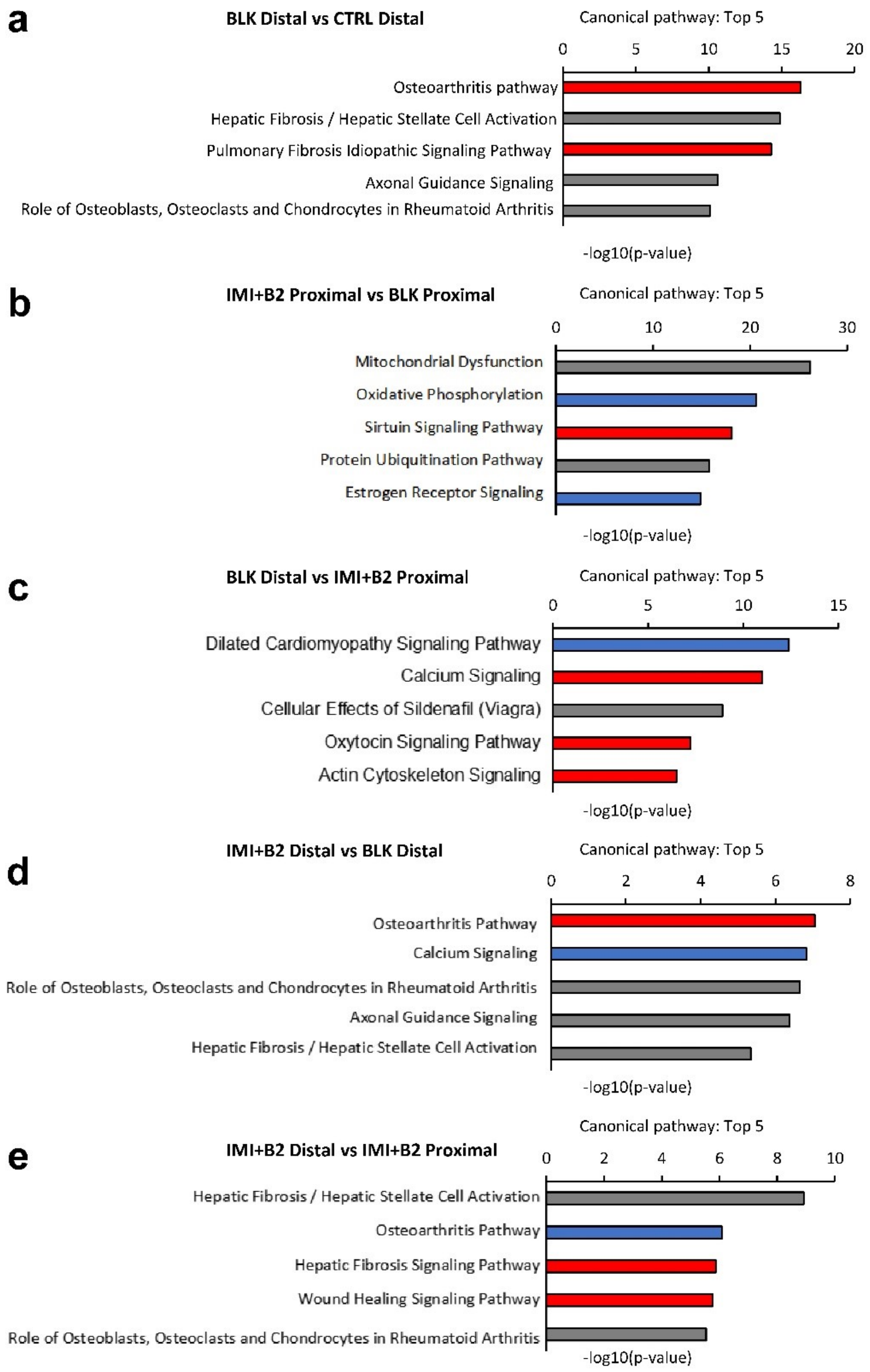

Fig. S20. Pathway affected by DO and (IMI+B2) during the early stage of bone regeneration. (a)-(e) Top 5 canonical pathway analysis curated by Ingenuity Pathways Analysis (IPA). Red bars indicate positive activation Z score; blue bars indicate negative activation Z score; gray bars indicate zero or unavailable activation Z score (n = 3 independent experiments per group). Data are analyzed with two-sided T-test with Bonferroni correction. Source data are provided as a Source data file.

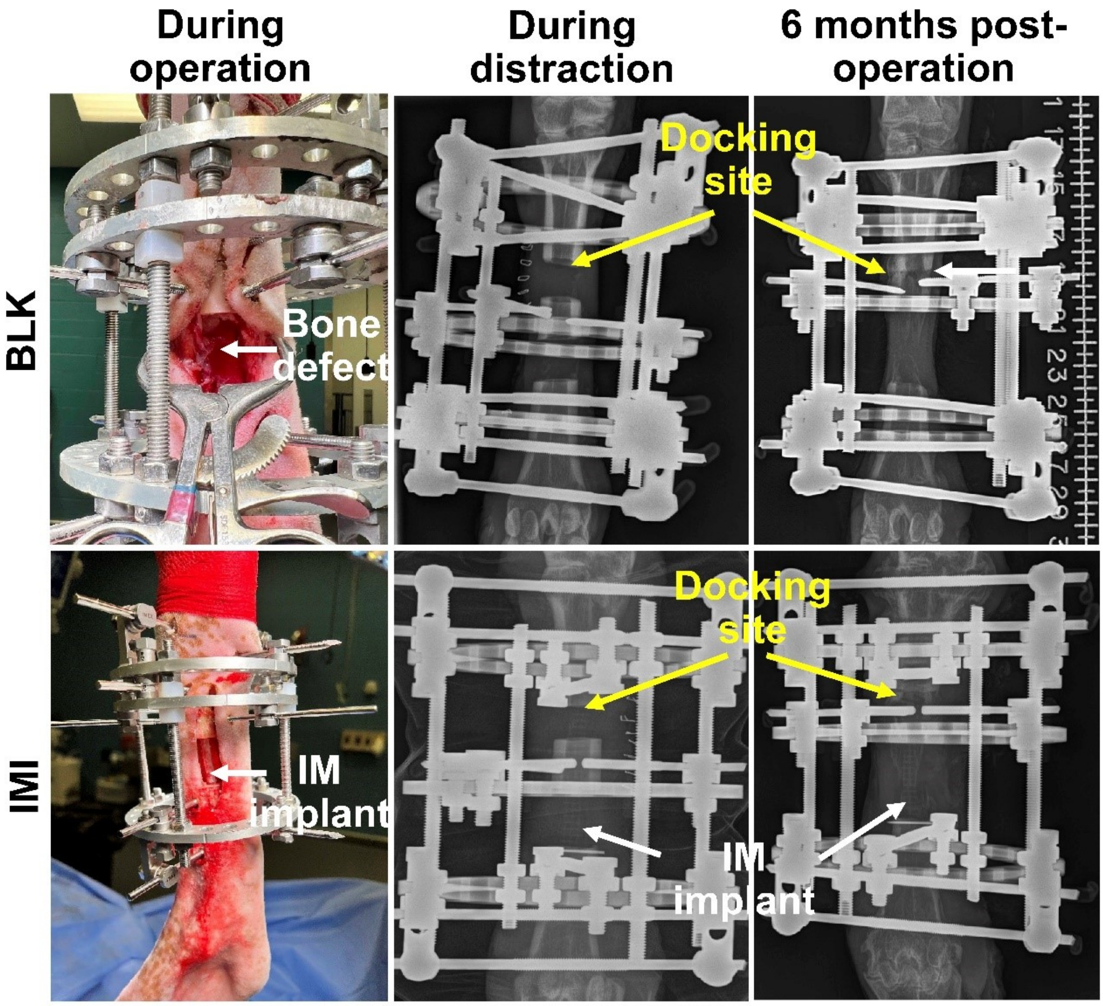

Fig. S21. Representative photos and radiographs of the sheep metatarsal bone transport model, distraction and device implantation. Photos at left column show the surgically created 30-mm bone defect in the metatarsus fix with circumferential fixators in the bone transport sheep model. The animals were treated with implantation of intramedullary implant (IMI) or without implant as blank control (BLK). The intercalary segments were transported retrogradely to the docking site (indicated by the yellow arrows). X ray images show the affected bone fix with circumferential fixators after 15-day distraction (middle column) or 6 months after implantation (right column).

Supplementary Tables

Table S1. Animal groups, treatments and the assessments in the study using rat bone transport model.

Groups	Treatments (Treated with bone transport)	Sample size & timepoints	X-ray, µCT, 3-point bending (n = 8)	Histomorphometry (n = 3)	Histology/ Immunohistochemistry (n = 5)	Gait (n = 2)
BLK	Blank (BLK) control, on implant	n = 8 (POD34) + 8 (POD55)	√	√	√	
CS + B2	Collagen sponge with BMP-2 (2 µg) at defect site	n = 8 (POD34) + 8 (POD55)	√	√	√	
IMI	IM implant (IMI) only, no BMP-2	n = 8 (POD34) + 8 (POD55)	√	√	√	√
IMI + B0.5	IMI + BMP-2 (0.5 µg)	n = 8 (POD34) + 8 (POD55)	√	√	√	
IMI + B2	IMI+ BMP-2 (2 µg)	n = 8 (POD34) + 8 (POD55)	√	√	√	√
IMI + B6	IMI + BMP-2 (6 µg)	n = 8 (POD34)	√	√	√	

Table S2. Pin tract infection details evaluated accordingly to a modified Checketts classification method by visual inspection.

**Grade**	**Time point**	**BLK**	**CS + B2**	**IMI**	**IMI + B0.5**	**IMI + B2**	**IMI + B6**
Grade 0	POD34	30/40	30/40	25/40	35/40	40/40	40/40
	POD55	20/40	25/40	20/40	35/40	40/40	NA
Grade 1	POD34	10/40	10/40	14/40	5/40	0/40	0/40
	POD55	17/40	11/40	16/40	5/40	0/40	NA
Grade 2	POD34	0/40	0/40	1/40	0/40	0/40	0/40
	POD55	3/40	4/40	4/40	0/40	0/40	NA

Note: Five pin-site in each rat, n = 8, e.g., a total of 40 pins per group per time point. Values are presented as the number of affected pins/ total number of pins in a group.
