# Supplementary material for: An osteoinductive and biodegradable intramedullary implant accelerates bone healing and mitigates complications of bone transport in male rats
Source: Nat Commun. 2023 Jul 24;14:4455. doi: 10.1038/s41467-023-40149-5 (PMC10366099; doi:10.1038/s41467-023-40149-5)
Supplement: Supplementary file 3 — Description of Additional Supplementary Files Document [file 41467_2023_40149_MOESM3_ESM.pdf]

Description of Additional Supplementary Files

Supplementary Movie 1: Gait performance of the rat in the IMI group on POD48 before fixator removal.

Supplementary Movie 2: Gait performance of the rat in the (IMI + B2) group on POD48 before fixator removal.

Supplementary Movie 3: Gait performance of the rat in the (IMI + B2) group on POD48 before fixator removal.

Supplementary Movie 4: Gait performance of the rat in the (IMI + B2) on POD55 after fixator removal.
