# Supplementary material for: An osteoinductive and biodegradable intramedullary implant accelerates bone healing and mitigates complications of bone transport in male rats
Source: Nat Commun. 2023 Jul 24;14:4455. doi: 10.1038/s41467-023-40149-5 (PMC10366099; doi:10.1038/s41467-023-40149-5)
Supplement: Supplementary file 8 — Reporting Summary [file 41467_2023_40149_MOESM8_ESM.pdf]

nature portfolio

Corresponding author(s): Yunzhi Peter Yang

Last updated by author(s): Jun 13, 2023

Reporting Summary

Nature Portfolio wishes to improve the reproducibility of the work that we publish. This form provides structure for consistency and transparency in reporting. For further information on Nature Portfolio policies, see our Editorial Policies and the Editorial Policy Checklist.

Statistics

For all statistical analyses, confirm that the following items are present in the figure legend, table legend, main text, or Methods section.

n/a	Confirmed	
☐	☒	The exact sample size (*n*) for each experimental group/condition, given as a discrete number and unit of measurement
☐	☒	A statement on whether measurements were taken from distinct samples or whether the same sample was measured repeatedly
☐	☒	The statistical test(s) used AND whether they are one- or two-sided<br>*Only common tests should be described solely by name; describe more complex techniques in the Methods section.*
☐	☒	A description of all covariates tested
☐	☒	A description of any assumptions or corrections, such as tests of normality and adjustment for multiple comparisons
☐	☒	A full description of the statistical parameters including central tendency (e.g. means) or other basic estimates (e.g. regression coefficient) AND variation (e.g. standard deviation) or associated estimates of uncertainty (e.g. confidence intervals)
☐	☒	For null hypothesis testing, the test statistic (e.g. *F*, *t*, *r*) with confidence intervals, effect sizes, degrees of freedom and *P* value noted<br>*Give P values as exact values whenever suitable.*
☒	☐	For Bayesian analysis, information on the choice of priors and Markov chain Monte Carlo settings
☒	☐	For hierarchical and complex designs, identification of the appropriate level for tests and full reporting of outcomes
☒	☐	Estimates of effect sizes (e.g. Cohen's *d*, Pearson's *r*), indicating how they were calculated

Our web collection on statistics for biologists contains articles on many of the points above.

Software and code

Policy information about availability of computer code

Data collection	Post processing of the micro-CT reconstructed images was analyzed using the SkyScan NRecon (version 1.7.4.6), CT Analyzer (CTAn; version 1.18.8.0) and CTVox (version 3.3.0 r1403), MicroView (version 2.5.0rc7). A material testing system (Instron 5944 testing system, Norwood, MA) with Bluehill software (Bluehill Universal, Instron) was applied in mechanical tests (Bluehill Universal is the version of the software). Histological images were taken under an All-in-One Fluorescence Microscope (version BZ-X800, Keyence, Osaka, Japan). Surface or interface pattern of the constructs were imaged using a low vacuum scanning electron microscope (Apreo S LoVac, Thermo Fisher Scientific) with Bruker Quantax EBSD 400i integrated system.
Data analysis	A priori power analysis (G*Power, Universität Düsseldorf) was used to evaluate sample size. All statistical analyses were performed using Prism 7.0 (GraphPad Software Inc.). RNA sequencing data analysis was performed using Transcriptome Analysis Console (TAC, Affymetrix, Santa Clara, CA) version 4.0 software. These data were normalized with MAS 5.0 algorithm (Affymetrix). Bacterial 16S-EZ sequencing data analysis was performed using using Quantitative Insights Into Microbial Ecology (Qiime, version 1.9.1) software.

For manuscripts utilizing custom algorithms or software that are central to the research but not yet described in published literature, software must be made available to editors and reviewers. We strongly encourage code deposition in a community repository (e.g. GitHub). See the Nature Portfolio guidelines for submitting code & software for further information.

Data

Policy information about availability of data

All manuscripts must include a data availability statement. This statement should provide the following information, where applicable:

- Accession codes, unique identifiers, or web links for publicly available datasets
- A description of any restrictions on data availability
- For clinical datasets or third party data, please ensure that the statement adheres to our policy

All the data are available in the main text or the supplementary materials. The microarray data are available in the GEO database under the accession numbers GEO: GSE200518 (https://www.ncbi.nlm.nih.gov/geo/query/acc.cgi?acc=GSE200518). The broad range of raw datasets acquired and analysed (or any subsets of it) are available from the corresponding author upon reasonable request.

Human research participants

Policy information about studies involving human research participants and Sex and Gender in Research.

Reporting on sex and gender	Not applicable.
Population characteristics	Not applicable.
Recruitment	Not applicable.
Ethics oversight	Not applicable.

Note that full information on the approval of the study protocol must also be provided in the manuscript.

Field-specific reporting

Please select the one below that is the best fit for your research. If you are not sure, read the appropriate sections before making your selection.

☒ Life sciences ☐ Behavioural & social sciences ☐ Ecological, evolutionary & environmental sciences

For a reference copy of the document with all sections, see nature.com/documents/nr-reporting-summary-flat.pdf

Life sciences study design

All studies must disclose on these points even when the disclosure is negative.

Sample size	A priori power analysis (G*Power, Universität Düsseldorf) based on the previous studies using a similar rat bone lengthening DO model in evaluating a bone graft or stem cell performances determined that a sample size of 8 was needed to obtain 90% statistic power at the significance value of 0.05 when comparing quantitative µCT analyses outcome. A reference reporting a similar DO rat model with the same sample size (n = 8) has been shown in the section of Statistical analysis. In the pilot pre-clinical sheep study, we have one sheep in each group (BLK and IMI groups).<br>In addition, sample size for microarray analysis (n = 3) was based on years of experiences in using bone tissue samples for DNA sequencing.
Data exclusions	No data were excluded from the analyses.
Replication	Data are representative of 2–3 independent experiments. We confirm that all the attempts at replications were successful. The number of repeats for each experiment and detailed descriptions of statistical tests are specified in the results section and the respective figure legends.
Randomization	Skeletal mature male Sprague Dawley (SD) rats or sheep were randomly assigned to different groups according to their body weights prior to any surgery or implantation.
Blinding	The assessments in the experiments were carried out using the same samples sequentially in a randomized and blinded fashion.

Reporting for specific materials, systems and methods

We require information from authors about some types of materials, experimental systems and methods used in many studies. Here, indicate whether each material, system or method listed is relevant to your study. If you are not sure if a list item applies to your research, read the appropriate section before selecting a response.

March 2021

Materials & experimental systems

n/a	Involved in the study
☐	☒ Antibodies
☒	☐ Eukaryotic cell lines
☒	☐ Palaeontology and archaeology
☐	☒ Animals and other organisms
☒	☐ Clinical data
☒	☐ Dual use research of concern

Methods

n/a	Involved in the study
☒	☐ ChIP-seq
☒	☐ Flow cytometry
☒	☐ MRI-based neuroimaging

Antibodies

Antibodies used	Immunohistochemistry was conducted by incubating the antibodies including anti-osteocalcin (sc-365797; Santa Cruz BioTech, Dallas, TX) in a dilution of 1:100, anti-CD31(sc-376764; Santa Cruz BioTech, Dallas, TX) in a dilution of 1:100, anti-BMP2 (ab6285; Abcam, Cambridge, UK) in a dilution of 1:200, or anti-VEGF antibody (ab1316; Abcam, Cambridge, UK) in a dilution of 1:100. The dilutions of antibodies have been updated in the revised manuscript.
Validation	The validation statements can be also found in manufacturers' or other reference website as follows:<br>sc-365797: https://datasheets.scbt.com/sc-365797.pdf<br>sc-376764: https://datasheets.scbt.com/sc-376764.pdf<br>ab6285: https://www.citeab.com/antibodies/714664-ab6285-anti-bmp2-antibody-65529-111<br>ab1316: https://www.abcam.com/products/primary-antibodies/vegfa-antibody-vg-1-ab1316.html?productWallTab=ShowAll<br>Each primary antibody has been validated before formal experiment. All of the antibodies in this study were proved to be functional and accurate in the paraffin-embedded rat samples when applied for immunohistochemistry (IHC).

Animals and other research organisms

Policy information about studies involving animals; ARRIVE guidelines recommended for reporting animal research, and Sex and Gender in Research

Laboratory animals	12-week-old male SD rats weighing 350 to 400 g were purchased from Charles River Laboratories. All surgeries were performed under anesthesia by 3 to 5% isoflurane-oxygen (VetOne, Boise, ID). Buprenorphine Sustained-Release (1 mg/kg; ZooPharm, Laramie, WY) and Carprofen (5 mg/kg; Zoetis, Parsippany, NJ) were administered subcutaneously to minimize the suffering of the animals before surgery and post-op for 3 days, respectively. Cefazolin (25 mg/kg; TCI, Tokyo, Japan) was also given subcutaneously post-op for 3 days. 8-mm femoral segment defect was surgically created and stabilized with the external fixator. Animals were housed individually after operation and fed with 2018 Teklad Global 18% Protein Rodent Diets (Teklad Diets, Madison, WI). Pin tract infection was monitored and managed in all the animals during the study period. Polyvinylpyrrolidone iodine and ethanol were used during pin tract care when required.<br>In addition, two skeletally mature Rambouillet Cross ewes aged three to four years old with a mean weight of 71 kg and a metatarsal length greater than 14 cm were selected. Under anesthesia, an optimized circular external fixator (IMEX, Longview, TX) was implanted on the right hind metatarsus. The sheep were monitored daily by veterinarians and qualified personnel for general health, neurologic problems, and lameness.
Wild animals	This study did not involve wild animals.
Reporting on sex	Only male SD rats or Rambouillet Cross ewes were used.
Field-collected samples	There is no field-collected samples in this study.
Ethics oversight	The animal experimental protocol for rat model was approved by the Institutional Animal Care and Use Committee (IACUC 33395) of Stanford University following ARRIVE guidelines. Approval for the surgeries on sheep was granted by the Colorado State Institutional Animal Care and Use Committee (approved protocol KP 1579). The ethical statement on sheep study has been updated in the revised manuscript.

Note that full information on the approval of the study protocol must also be provided in the manuscript.
